# Latitudinal gradients in sexual dimorphism: Alternative hypotheses for variation in male traits

**DOI:** 10.1002/ece3.8386

**Published:** 2021-11-30

**Authors:** Christopher M. Murray, Caleb D. McMahan, Allison R. Litmer, Jeffrey M. Goessling, Dustin Siegel

**Affiliations:** ^1^ Department of Biological Sciences Southeastern Louisiana University Hammond Louisina USA; ^2^ Field Museum of Natural History Chicago Illinois USA; ^3^ Department of Biology University of Arkansas Fayetteville Arkansas USA; ^4^ Natural Sciences Collegium Eckerd College, Saint Petersburg Florida USA; ^5^ Department of Biology Southeast Missouri State University Cape Girardeau Missouri USA

**Keywords:** female‐choice system, gradient, latitude, sexual dimorphism, sexual selection

## Abstract

Biological patterns across latitudinal gradients elucidate a number of striking natural clines from which numerous processes can be further explored. The trade‐off between reproduction and somatic maintenance and growth represents a suite of life‐history traits with variable energy allocation and potential latitudinal patterns. Specifically, male sexually dimorphic traits in female choice systems represent one such reproductive investment constrained by resource acquisition and subsequent allocation. Latitudinal variation in sexual dimorphism has been suggested although the relationship between dimorphic traits and latitude are conflicting. Here, we test alternative hypotheses regarding this pattern using two broadly distributed vertebrates exhibiting sexually dimorphic traits. We hypothesized that the exaggeration of dimorphic traits correlates with latitude, with males having exaggerated sexually dimorphic traits at either higher or lower latitudes. Results indicate that male sexually dimorphic traits are exaggerated at lower latitudes while relative gonopodium size in *Poecilia latipinna* was larger at higher latitudes. This pattern may be a result of lower latitude populations experiencing greater population densities and longer access to resources that could manifest in females more intensively selecting for higher quality males in lower latitudes. Experimental work should address this pattern and investigate mechanistic processes.

## Graphical abstract

We test the hypothesis that the exaggeration of sexually dimorphic traits correlates with latitude, with males having exaggerated sexually dimorphic traits at either higher or lower latitudes. Results indicate that male sexually dimorphic traits are exaggerated at lower latitudes. Experimental work should address this pattern and investigate mechanistic processes.

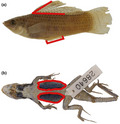

## 1 INTRODUCTION

The study of biological processes across latitudinal gradients has uncovered one of the most robust natural clines from which ecological and biogeographic patterns can be deduced. In general, diversity itself is at its peak near the equator and declines at higher latitudes (i.e., the latitudinal diversity gradient; Fischer, 1960). Explanations for this pattern are numerous and uncover other latitudinal trends, such as the relative predominance of ecological interactions (i.e., mutualisms and complex predation networks; Janzen, 1966; Schemske et al., 2009). Furthermore, Rapoport's rule (Rapaport, 1982; Rapoport, 1975) and Bergman's rule (Bergmann, 1848; Blackburn et al., 1999) describe long‐standing latitudinal patterns in geographic range size and homeothermic body size, respectively. Ecologically, species‐specific niche breadth is thought to increase at higher latitudes (MacArthur, 1972; Vázquez & Stevens, 2004), and even population dynamics (Turchin & Hanski, 1997) and density‐dependent mortality (Lambers et al., 2002) are thought to exist on latitudinal gradients. Some traits, such as thermoregulatory physiology in bats (Dunbar & Brigham, 2010) and crabs (Gaitán‐Espitia et al., 2014), vary intraspecifically and across latitudinal distribution as a function of changes in energy allocation within individual organisms in the presence of different environmental attributes.

Cost of reproduction versus cost of somatic maintenance and growth represent one such suite of life‐history traits with variable energy allocation among individuals based on environmental attributes (Glazier, 1999). Differential energy allocation (reproductive versus somatic) occurs when resource acquisition varies relative to the total resources necessary (Glazier, 1999). Such trade‐offs manifest in negative relationships between reproduction and other critical processes, including immune response (Adamo et al., 2001), stress response (Petes et al., 2008), longevity (Kaitala, 1991), growth (Roff, 2000), and dispersal (Hughes et al., 2003). Reproductive investments themselves can vary in the somatic trade‐off. The most obvious response variables for reproductive allocation (or lack there‐of) are egg/offspring size (Murray et al., 2013), number (Boyce & Perrins, 1987), and frequency of reproductive events (Christie et al., 2018). However, reproductive investment depends on a variety of individual and environmental factors, such as sex, mating system, and resource availability.

Sexually dimorphic traits represent one such reproductive investment that is constrained by resource acquisition (Reichard et al., 2008). Sexual dimorphism between males and females is hypothesized to have evolved in response to sexual selection pressures and/or intraspecific niche divergence (Shine, 1989). Reasons for sexual dimorphism include a selected increase in male body size for male–male combat (Darwin, 1874), female‐choice (Kirkpatrick, 1982), or sex‐specific niche divergence (Shine, 1989). As noted, dimorphism in body size is observed frequently in nature (e.g., Price, 1984); however, many sexually dimorphic traits are not size based. Color (Price & Birch, 1996), ornamental structures (Kraaijeveld, 2019), behaviors (Pruett‐Jones & Pruett‐Jones, 1990), and chemical cues (Parker & Mason, 2012) present other notable examples of sexual differences, all of which are subject to resource‐based variation. Badyaev (1997) documents elevational clines in male plumage potentially based on resource constraints and energy diversion to bi‐parental care. Other such patterns have been documented along temporal clines during warming periods (Post et al., 1999).

Latitudinal variation in sexual dimorphism has been suggested interspecifically (Wallace, 1878), with species at lower latitudes having exaggerated dimorphism due to more pronounced sexual selection relative to species at higher latitudes (Sumarto et al., 2020), although this conclusion is not unanimous among clades (Fujimoto et al., 2015). Within species, Lima‐Filho et al. (2017) recovered reduced sexual dimorphism in size and shape at higher latitudes when comparing five discrete populations of goby fish along the Brazilian coast. These results, however, were more closely attributed to population‐specific pressures as opposed to a gradient‐like pattern, but the relationship between male sexual dimorphism and latitude remains ambiguous both inter‐ and intraspecifically.

In theory, because resources and sexual selection varies latitudinally, the interaction between resource‐dependent reproductive energy allocation and the sexual selection pressures presents a situation in which intraspecific variation in sexual dimorphism may exist along a latitudinal cline. Such a cline poses a gradient in population density, resource availability, and seasonality. If sexual selection predominantly drives intraspecific sexual dimorphism, then latitude and the prominence of sexually dimorphic traits may be positively associated, and dimorphic traits would be exaggerated where the breeding season is short and resources are limited (i.e., higher latitudes). Conversely, if resource allocation or population density predominantly drives intraspecific sexual dimorphism, then latitude and the prominence of sexually dimorphic traits may be negatively associated. Here, we test for these alternative patterns. We hypothesize that the exaggeration of dimorphic traits correlates with latitude, with males having exaggerated sexually dimorphic traits at either higher latitudes with seasonality and lower resource availability or, alternatively, lower latitudes with less seasonality and higher resource availability. We test these hypotheses using two broadly distributed and unrelated North American vertebrates exhibiting sexually dimorphic traits and comment on potential mechanisms responsible for observed patterns.

## 2 METHODS

### 2.1 Taxon sampling and trait data

Fifty‐eight (58) adult male Sailfin Molly specimens (*Poecilia latipinna*) were used for this study (Appendix 1). Males of this species have an enlarged sexually dimorphic dorsal‐fin used to solicit female choice in mating (Robins et al., 2018). The presence of a well‐developed gonopodium, the intromittent reproductive organ in poeciliid fishes, determined reproductive maturity. Only individuals above 2.0 cm standard length were used, as indicated by histological verification of size at maturity (Robins et al., 2018; Snelson, 1985). Specimens ranged in latitude from 24.55°N to 34.52°N.

One hundred one (adult male Eastern Fence Lizard specimens (*Sceloporus undulatus*) were analyzed (Appendix 1). Males of this species have a sexually dimorphic, brightly colored venter used in mating display to solicit female choice in mating (Cooper & Burns, 1987). Reproductive maturity was determined by observation of enlarged postanal scales (Cox & John‐Alder, 2007). Specimens ranged in latitude from 20.85°N to 41.71°N.

**FIGURE 1:**
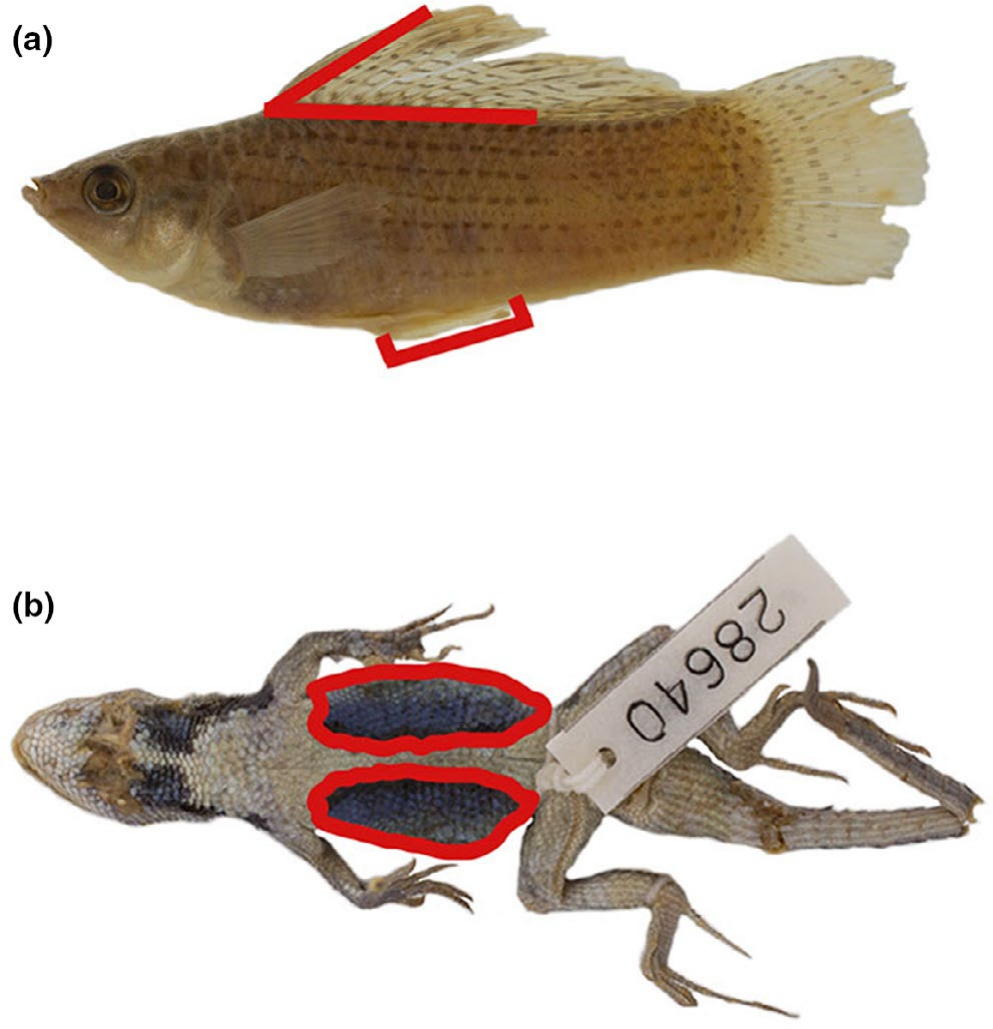
Focal species highlighting the sexually dimorphic traits quantified for this study. (a) dorsal‐fin height, dorsal‐fin length and gonopodium length in *Poecilia latipinna*; (b) proportion of venter with pigment in *Sceloporus undulatus*

For specimens of *Poecilia latipinna*, gonopodium length, dorsal‐fin height, dorsal‐fin length, and standard length were measured using digital calipers to the nearest 0.1 mm (Figure 1a). Gonopodium length, dorsal‐fin height, and dorsal‐fin length were standardized (divided by standard length) to account for allometry. *Sceloporus undulatus* specimens were photographed using a Canon DSLR on an illuminated camera stage and photographs were imported into ImageJ (Schneider et al., 2012). The total surface area of the venter and the surface area of the sexually dimorphic pigmented venter patches were calculated in ImageJ and the colored area divided by the total venter surface area to calculate the proportion of the venter that was pigmented (Figure 1b). It is important to note that surface area of the venter patch does not vary with seasonal reproductive condition, but rather with body size for which we standardize (Lemos‐Espinal et al., 1996).

### 2.2 Analysis

Individual generalized additive models (GAM) were used to assess the relationship between latitude and standardized gonopodium length, standardized dorsal‐fin length, and standardized dorsal‐fin height for *P*. *latipinna*, and latitude and proportion of venter with pigment for *S*. *undulatus*. All GAMs were performed using R Statistical Software (R Core Team, 2018) with the package “mgcv” (Wood, 2011).

## 3 RESULTS

**FIGURE 2:**
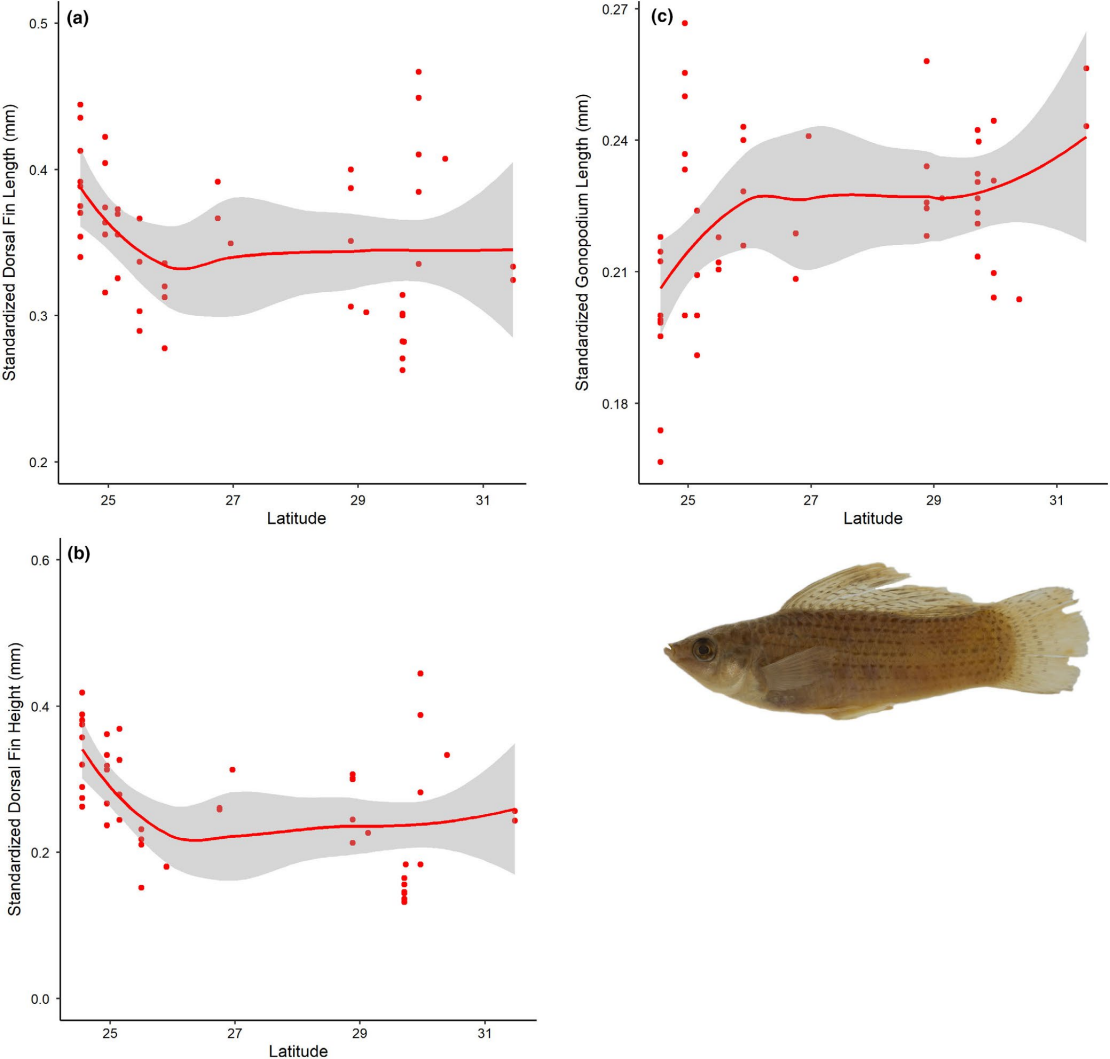
Generalized Additive Model depicting the relationships between male sexually dimorphic traits and latitude in *Poecilia latipinna*. (a) A decrease in dorsal‐fin height with increasing latitude, (b) a decrease in dorsal‐fin length with increasing latitude, (c) an increase in gonopodium length at with increasing latitude

**FIGURE 3:**
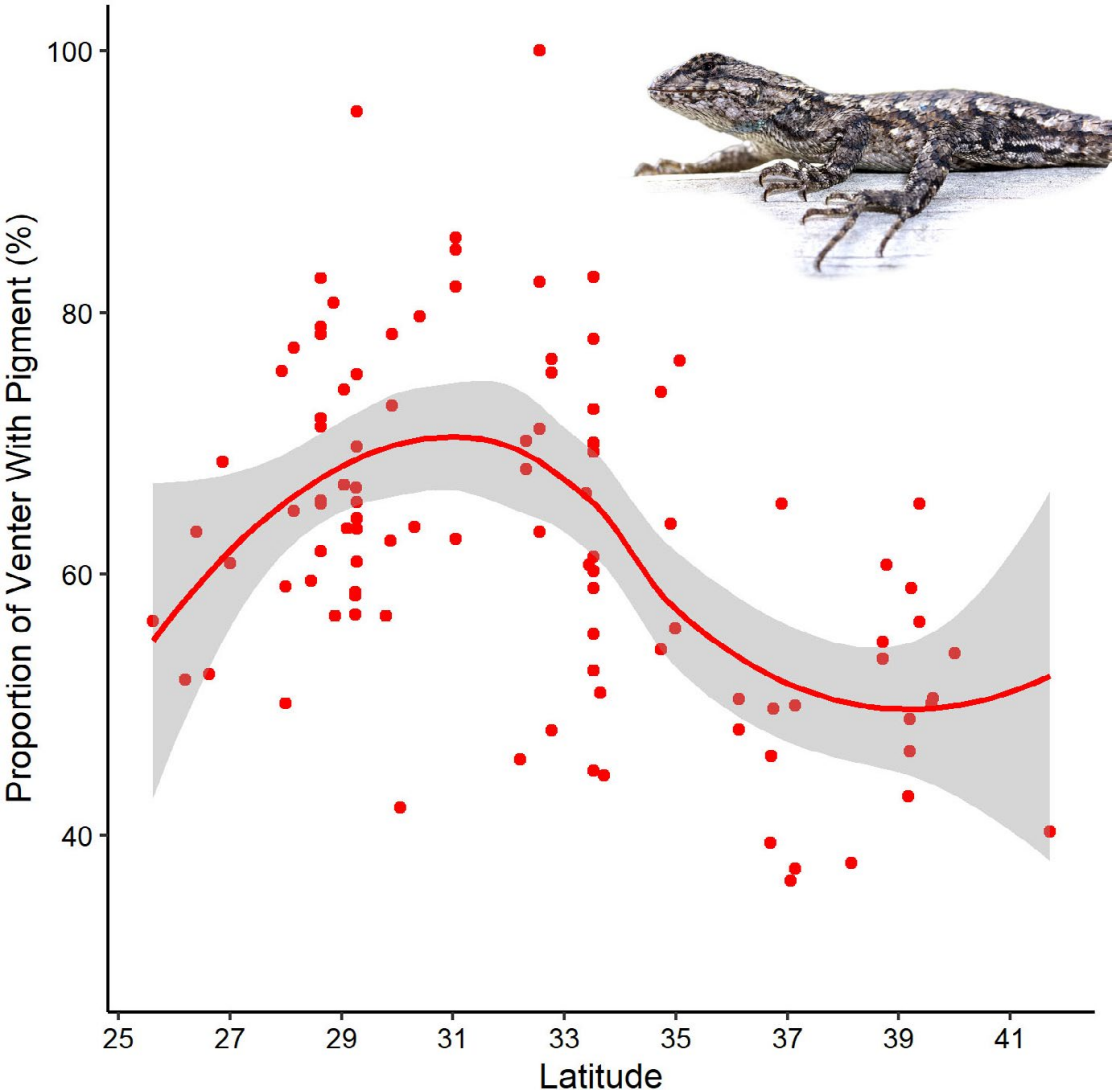
Generalized Additive Model depicting the negative relationship between male sexually dimorphic venter coloration and latitude in *Sceloporus undulatus*

Standardized gonopodium length for *P*. *latipinna* increased significantly with latitude (df = 8.674, *f* = 3.118, *p* = .004; *R* ^2^ = 0.309; Figure 2c). Dorsal‐fin height and length also varied significantly with latitude; however, dorsal‐fin height (df = 8.708, *f* = 8.157, *p* = <.001; *R* ^2^ = 0.569; Figure 2b) and dorsal‐fin length (df = 8.874, *f* = 5.868, *p* = <.001; *R* ^2^ = 0.482; Figure 2a) were larger at lower latitudes. The proportion of the venter with pigment in *S*. *undulatus* also varied significantly across latitude (df = 4.968, *f* = 9.015, *p* = <.001; *R* ^2^ = 0.306; Figure 3) with the highest proportion of venter with pigment around lower latitudes, but not the lowest.

## 4 DISCUSSION

Our results demonstrate a significant and indirect correlation when comparing latitude to dorsal‐fin height and length in male *P*. *latipinna*. This supports our alternative pattern hypothesis that sexually dimorphic traits are exaggerated at lower latitudes relative to higher latitudes. Female *P*. *latipinna* prefer male conspecifics with larger dorsal fins (MacLaren et al., 2004) used in courtship displays (Farr et al., 1986; Ptacek & Travis, 1996). Mechanistically, a longer active season and/or higher density at lower latitudes may provide more resources and drive competition within males to increase the perceived structural quality.

Increased dimorphism in the male dorsal‐fin traits in lower latitudes may indicate that intrasexual competition itself is more intense in lower latitudes. Population densities likely decrease with increased latitude (and away from the species core; Schemske et al., 2009), thus male–male interactions can increase in lower latitudes where competition for mates is greater. In higher latitudes, population fitness is more impacted by abiotic environmental constraints (Briscoe Runquist et al., 2020), whereas in lower latitudes fitness is most impacted by biotic interactions (such as competition, predation, and parasites; Gaston, 2003; Schemske et al., 2009; Wisz et al., 2013). Tropical populations interact with more species and show faster rates of adaptation in response to biotic pressures, whereas temperate populations are principally constrained by abiotic pressures on fitness (Schemske, 2009; Schemske et al., 2009). Because biotic interactions are more important in shaping species distributions in lower latitudes (Schemske et al., 2009), it is possible that a cline in population density has also driven the dimorphism of sexually selected traits in males. In this case, males in lower latitudes likely experience greater population densities, consistent with populations in the core of a distribution, and thus males are under more intense selection for ornamentation. In more temperate latitudes, where population density is likely lower and abiotic, not biotic, parameters constrain male fitness, males are released from intense selection for elaborate ornamentations and instead evolve morphological traits best suited to the respective abiotic environment.

We recovered the opposite pattern when comparing latitude to gonopodium length in *P*. *latipinna* (i.e., a significant direct correlation), with males possessing longer gonopodia at higher latitudes. This pattern result supports an alternative process hypothesis that sexually dimorphic traits would be exaggerated where the breeding season is short and resources are limited. An elongated gonopodium increases the chance that a male *P*. *latipinna* successfully inseminates a female by allowing a male to better visualize the tip of the gonopodium when approaching the gonopore of a female. This type of copulation is termed “sneak copulation” because males use the gonopodia to sneak up on females for insemination (Houde, 1997). Thus, males with longer gonopodia are more efficient at sneak copulation versus males with shorter gonopodia (Greven, 2005). When resources and breeding season are more limited by latitude, intersexual selection could drive resource allocation toward secondary sexual structures that benefit individuals during “sneak” intersexual competition.

When comparing gonopodia across poeciliid species, mating tactics vary depending on length of the gonopodium. In species where females predominantly choose displaying males, male gonopodia tend to be less than a third of the length of the body; whereas, in species where males do not display, gonopodia longer than a third of the length of the body are more prominent (Greven, 2005). The prior condition where females choose a male based on ornamentation associated with courtship display is more successful in procuring a female mate in poeciliids (Pilastro & Bisazza, 1999). Our results provide correlative evidence that differential resource allocation may be the driving force for this mating tactic variation. Whereas preferred male phenotypes (e.g., ornamentations and courtship display) are typically costly in terms of energy allocation, alternate mating tactics (e.g., sneaker males) are relatively cheaper energetically (Cummings & Gelineau‐Kattner, 2009). It can be expected that many species follow this same pattern, where shorter active seasons at higher latitudes drive increased sexual selection for sperm production/transfer mechanisms versus mechanisms to attract a female.

Similar to dorsal‐fin height and length in *P*. *latipinna*, the total surface area covered in pigmented blue scales was greater in *S*. *undulatus* from more southern latitudes; that is, a significant negative correlation with latitude. Although there is no evidence that patch morphology co‐varies with fitness in *S*. *undulatus*, “bluer” males were more likely to sire at least one offspring in a recent study (Robinson et al., 2021). Alternatively, male cost of reproduction for lizards has been quantified by measuring changes in body mass (Abell, 2000) and a decrease in consumption (Weiss, 2001) throughout the breeding season. However, considering “blueness” is directly correlated with testosterone levels in *S*. *undulatus* (Robinson et al., 2021), it is obvious that the blue patch can be considered with other reproductive traits when considering resource allocation; unfortunately, its role in reproduction is unknown (Robinson et al., 2021). Because patches or ornaments are typically associated with female choice mating system (Andersson, 1982), we consider the trend of more pigmented *S*. *undulatus* males at lower latitudes to support our hypothesis that intersexual selection drives resource allocation toward secondary sexual structures for attracting a mate in female choice mating systems when resources are less limited by a shorter active season. And, as described above for male dorsal fins in *P*. *latipinna*, southern populations also likely exhibit greater population densities, and thus increased competition for mates among males, thereby driving selection of key secondary sex characters that are selected by females.

Here, we elucidate repeated patterns between male traits in female‐choice systems among distantly related taxa. Langerhans et al. (2005) found that female *Gambusia* prefer males with longer gonopodia, but the presence of predators reverses the direction of selection and drives a reduction in intromittent organ size. Thus, the opposite pattern of this trait compared to dorsal‐fin traits likely reflects trade‐offs in different directions between female choice and more complex natural selection (including survival). Importantly, the traits that are more directly under female choice (i.e., dorsal‐fin size in *P*. *latipinna* and blueness in *S*. *undulatus*) showed a negative relationship with latitude, while the traits less under female influence in *P*. *latipinna* (i.e., gonopodium length) showed an opposite pattern. These results demonstrate that female choice is a more potent driver of dimorphism in lower latitudes. The presence of this pattern, however, does not by itself reveal the mechanism behind which intensity of female choice varies latitudinally. Understanding the mechanism behind these patterns requires experimental assessment (such as reciprocal translocation and/or controlled density experiments) to isolate the naturally confounded effects of resource availability and population density. That is, lower latitude populations likely experience both greater population densities and longer access to more resources; both these mechanisms could manifest as females more intensively selecting for “higher quality” males in lower latitudes. Future work should address exaggerated sexual dimorphisms at lower latitudes, both investigating the process and corroborating the pattern.

## CONFLICT OF INTEREST

The authors have no conflict of interest to declare.

## AUTHOR CONTRIBUTIONS

**Christopher M. Murray:** Conceptualization (equal); data curation (equal); formal analysis (equal); investigation (equal); methodology (equal); project administration (equal); resources (equal); supervision (equal); visualization (equal); writing–original draft (equal); writing–review and editing (equal). **Caleb D. McMahan:** Conceptualization (equal); data curation (equal); formal analysis (equal); methodology (equal); project administration (equal); visualization (equal); writing–original draft (equal); writing–review and editing (equal). **Allison Litmer:** Formal analysis (equal); methodology (equal); writing–original draft (equal); writing–review and editing (equal). **Jeffrey Goessling:** Methodology (equal); resources (equal); validation (equal); writing–original draft (equal); writing–review and editing (equal). **Dustin Siegel:** Conceptualization (equal); data curation (equal); methodology (equal); project administration (equal); visualization (equal); writing–original draft (equal); writing–review and editing (equal).

## Data Availability

All data associated with the analyses in this manuscript are archived in Dryad: https://doi.org/10.5061/dryad.cvdncjt5d.

## APPENDIX 1

Catalog numbers for museum specimens used in the study. FMNH = Field Museum of Natural History, NCSM = North Carolina Museum of Natural Science.

***Poecilia latipinna*: FMNH (54)**: 78611, 13102, 87215, 113099, 7004, 81925, 38396, 50532, 90907, 128723, 13988, 51052, 83648, 128702, 50707, 90966. **NCSM (4):** 74560, 65554, 31365

***Sceloporus undulatus*: FMNH (103)**: 1000, 1001, 1340, 11955, 17046, 17048, 18018, 21468, 21499, 26877, 27391, 27392, 27396, 27397, 27398, 27400, 27402, 27404, 28492, 28635, 28638, 28640, 29463, 29464, 33247, 33251, 33252, 33255, 33256, 33257, 33499, 33965, 33966, 33968, 33969, 33970, 33971, 33972, 38193, 38194, 41847, 42381, 46071, 47191, 51856, 56488, 62833, 62834, 94972, 94982, 94984, 95977, 95978, 95980, 106220, 106257, 106530, 106531, 106969, 106972, 106973, 108672, 112168, 112169, 112173, 125199, 126277, 126280, 126285, 194390, 194390, 208108, 208109, 208111, 208114, 213575, 240453, 240455, 240456, 240457, 240458, 240461

240462, 240465, 240466, 240467, 240468, 240470, 247113, 247114, 247116, 247118

247119, 247123, 247125, 1000‐2, 1000‐3, 1000‐4, 1000‐5, 1000‐6, 1000‐7, 1340‐2

17046‐2
